# Long Non-Coding RNA *HOTAIR* in Breast Cancer Therapy

**DOI:** 10.3390/cancers12051197

**Published:** 2020-05-09

**Authors:** Monica Cantile, Maurizio Di Bonito, Margherita Cerrone, Francesca Collina, Michelino De Laurentiis, Gerardo Botti

**Affiliations:** 1 Pathology Unit, Istituto Nazionale Tumori-Irccs-Fondazione G.Pascale, 80131 Naples, Italy; m.dibonito@istitutotumori.na.it (M.D.B.); margherita.cerrone@istitutotumori.na.it (M.C.); f.collina@istitutotumori.na.it (F.C.); 2 Breast Unit, Istituto Nazionale Tumori-Irccs-Fondazione G.Pascale, 80131 Naples, Italy; m.delaurentiis@istitutotumori.na.it; 3 Scientific Direction, Istituto Nazionale Tumori-Irccs-Fondazione G.Pascale, 80131 Naples, Italy; g.botti@istitutotumori.na.it

**Keywords:** breast cancer, lncRNA *HOTAIR*, drug resistance, breast cancer therapy

## Abstract

Breast cancer (BC) is the most common cancer type among women, and morbidity and mortality rates are still very high. Despite new innovative therapeutic approaches for all BC molecular subtypes, the discovery of new molecular biomarkers involved in tumor progression has been fundamental for the implementation of personalized treatment strategies and improvement of patient management. Many experimental studies indicate that long non-coding RNAs (lncRNAs) are strongly involved in BC initiation, metastatic progression, and drug resistance. In particular, aberrant expression of HOX transcript antisense intergenic RNA (*HOTAIR*) lncRNA plays an important role in BC contributing to its progression and represents a predictor of BC metastasis. For its proven prognostic value, *HOTAIR* could represent a potential therapeutic target in BC. In the present review, we summarize the role of HOTAIR in cancer progression and drug resistance, in particular in BC, and we illustrate the main approaches for silencing it.

## 1. Introduction

Breast cancer (BC) is the most prevalent cancer type in women and a leading cause of cancer mortality in the world. Breast cancer is a very heterogeneous disease, and its histological classification is mainly based on the expression of hormonal receptors such as estrogen receptor (ER), progesterone receptor (PR), and ERBB2 receptor (HER2) [1]. With respect to gene expression, BC is classified into five molecular subtypes including luminal ER positive (luminal A and luminal B), HER2 enriched, basal-like (also known as triple-negative breast cancer), and normal breast-like subtype [2]. Currently, the choice of routine treatment strategy is based on various factors including tumor size, morphology, grade, metastases, and ER, PR, and HER2 expression [3]. In the last ten years, new innovative therapeutic approaches have been optimized, in particular for triple-negative breast cancer [4]. However, the identification of other prognostic/predictive markers is fundamental for implementing personalized treatment strategies in BC. In this context, our understanding of the mechanisms that regulate gene expression has focused on a class of non-coding RNA molecules (ncRNAs) which have aberrant activity that has largely been described in BC tumor progression [5].

Long non-coding RNAs (lncRNAs) represent a class of ncRNAs, longer than 200 nucleotides, involved in various aspects of cellular homeostasis, such as proliferation, apoptosis, mobility, gene transcription, and post-transcriptional processing [6,7,8]. They can be classified into different categories based on their genomic position, subcellular localization, and function [8]. Regarding their location in the genome, lncRNAs are classified into sense, antisense, bidirectional, and intergenic and intronic lncRNAs, while according to their subcellular location, lncRNAs are classified as nuclear lncRNAs and cytoplasmic lncRNAs. The identification of their precise cellular sub-localization is fundamental to understanding their cellular activity [9]. Long non-coding RNAs are essential epigenetic regulators of transcription functioning as: (i) molecular signals to regulate transcription in response to various stimuli [10]; (ii) decoys, modulating the transcription by sequestering regulatory factors and reducing their availability [11]; (iii) scaffolds, playing a structural role as platforms for the assembly of multiple-component complexes such as ribonucleoprotein (RNP) complexes [12]; (iv) enhancer RNAs, influencing the three-dimensional (3D) organization of DNA (chromatin interactions) [13]; (v) short peptides coders which may also interfere with transcription [14].

Long non-coding RNA’s role in cancer has been widely described, highlighting their capability to influence cell cycle regulation, cell proliferation, trans-differentiation, survival, immune response, metastatic progression, and therapeutic response [15]. Moreover, many lncRNAs are transcriptionally regulated by key tumor suppressors or oncogenes [16,17]. In cancer, lncRNAs are mainly involved in chromatin remodeling [18]. They can directly interact with many histone and DNA-modifying enzymes to participate in covalent modifications of histones or DNA. Furthermore, several lncRNAs have recently been found to be capable of modulating the non-covalent, ATP-dependent chromatin remodeling process, indicating an extensive role of lncRNAs in chromatin regulation.

Being LncRNAs expressed in a specific manner in a type of cancer and regulating fundamental processes during tumor progression, they could represent not only exceptional diagnostic, prognostic, and predictive markers but also potential therapeutic targets. Many lncRNAs have been associated with BC, and most of them interfere with crucial processes during BC carcinogenesis [19,20].

In this review, we will discuss the role of the lncRNA *HOTAIR* in BC, highlighting in particular its contribution to tumor progression and drug resistance mechanisms and suggesting its potential use as therapeutic target.

## 2. LncRNAs in Breast Cancer

Long non-coding RNAs play a main role in BC tumor progression (Table 1). They are able of inducing the metastatic process by modulating cell proliferation, invasion, migration, epithelial–mesenchymal transition (EMT), and self-renewal capacity. Clinically, many lncRNAs are involved in therapeutic sensitivity, and they are becoming important circulating biomarkers [5,21,22].

Among the lncRNAs involved in BC evolution, *H19* is one of the most studied. Its aberrant expression is associated with an increased risk of BC, both in human and cell models [23]. Moreover, its detection in plasma of BC patients also suggests its use as a circulating marker [24]. The expression level of *H19* is associated with tumor size, lymph nodes status, and poor prognosis, especially in triple-negative BC (TNBC) [25]. Furthermore, the overexpression of *H19* is able to induce chemotherapy resistance in BC cells and its silencing sensitizes BC endocrine therapy resistance (ETR) cells to tamoxifen and fulvestran treatment [26,27]. Long non-coding RNA *XIST* (X inactive specific transcript) is strongly associated with BC evolution, and it is able to suppress BC cell growth, migration, and invasion via the miR-155/CDX1 axis [28]. Aberrant expression of *BCAR4* (breast cancer anti-estrogen resistance 4) is mainly involved in acquiring BC tamoxifen resistance [29] in an independent manner of estrogen receptor I (ESRI) [30]. In addition, *BCAR4* is able to promote metastasis through the interaction with chemokine CCL21 and its receptor CXCR7 in BC cell models [31]. Colon cancer-associated transcript 2 (*CCAT2*) is overexpressed, in particular, in TNBC cells, in which it is able to promote cell proliferation, migration, and invasion. In addition, aberrant expression of *CCAT2* significantly induces stem-like characteristics in TNBC cells [32]. Urothelial carcinoma associated 1 (*UCA1*) is upregulated in tamoxifen-resistant BC cells [33], and its knockdown reduces cell survival and migration ability and promotes apoptosis of tamoxifen-resistant BC cells [34]. The role of lncRNA *MALAT1* (metastasis-associated lung adenocarcinoma transcript 1) in BC has been widely discussed. Many studies suggested its role as a metastasis-promoting marker [35], but other in vitro and xenograft studies have highlighted contradictory effects on BC tumor cells [36]. A recent genetic study has showed that *MALAT1* is able to bind and inactive *TEAD* (TEA domain transcription factor 1), a pro-metastatic transcription factor, and consequently suppresses BC metastasis [37]. Nuclear enriched abundant transcript 1 (*NEAT1*) is another lncRNA involved in breast gland development, and it has been associated with BC evolution. It is able to promote proliferation and progression in BC cells [38]. Nuclear enriched abundant transcript overexpression is associated with tumor size, histological grade, metastasis, and poor survival [39]. Most of the other lncRNAs described in the literature are mainly associated with therapeutic resistance in BC. The upregulation of lncRNA-*ATB* (long non-coding RNA activated by TGF-Beta) [40], *TINCR* (Tissue differentiation-inducing non-protein coding RNA) [41], *UCA1* [42], *AGAP2-ASI* (Arf GAP with GTP-binding protein-like domain, Ankyrin repeat, and PH domain 2) [43], and the downregulation of *GAS5* (growth arrest-specific 5) [44] are strongly involved in acquiring trastuzumab resistance in BC patients. The upregulation of *BCAR4*, *UCA1*, and *CCAT2*, as previously indicated, together with the aberrant expression of lncRNA-*ROR* (regulator of reprogramming) [45], lncRNA uc.57 [46], *LINP1* (LncRNA in non-homologous end-joining pathway 1) [47], *DSCAM-ASI* (Down syndrome cell adhesion molecule-antisense RNA 1) [48], *ADAMTS9-AS2* (ADAM metallopeptidase with thrombospondin Type 1 motif 9-antisense RNA 2) [49], *CyTOR* (cytoskeleton regulator RNA) [50], and the downregulation of *GAS5* [51] are involved in the promotion resistance mechanisms of tamoxifen and chemotherapy [34].

## 3. LncRNA HOTAIR and Its Role in Cancer

HOX transcript antisense RNA (*HOTAIR*) is an lncRNA 2158 bp long, consisting of 6 exones, located on chromosome 12q13.13 between *HOXC11* and *HOXC12* genes [52]. Its promoter contains binding sites for many transcription factors, such as AP1, Sp1, ERE elements, HRE elements, and NF-kB [53]. HOX transcript antisense RNA (*HOTAIR*) is a key regulator of chromatin status and a mediator of transcriptional silencing [53]. Early studies showed that *HOTAIR* is capable to bind the *PRC2* (Polycomb repressive complex) at the 5′ end [52]. The formation of the molecular complex is able to maintain cell stemness and suppress cell differentiation by trimethylation of the H3K27 histone complex and subsequent transcriptional repression of differentiation genes [53]. HOX transcript antisense RNA (*HOTAIR*) is also able to interact at the 3′ end with the lysine-specific histone demethylase 1A (*LSD1*), another chromatin modifier which is critical for gene silencing [54]. Lysine-specific histone demethylase 1A (*LSD1*) can form a multiprotein complex via activation of RE1-silencing transcription factor (REST) and CoREST which are critical players in gene silencing [54]. HOX transcript antisense RNA (*HOTAIR*) acts as a molecular scaffold for the conjunction of the two complexes. The HOTAIR-PRC2-LSD1 complex leads epigenetic changes contributing to the targeted gene silencing and represses their transcription via H3K27 trimethylation (PRC2 activity) and H3K4 demethylation (LSD1 activity). For example, the HOTAIR-PRC2-LSD1 complex can be redirected towards the 5′ end of the HOXD locus on chromosome 2 where the genes, implicated in metastatic suppression are silenced by methylation and demethylation of H3K27 and H3K4, respectively [52,55].

HOX transcript antisense RNA (*HOTAIR*) can also alter gene expression both at the post-transcriptional level, either by base pairing with translation factors or ribosomes to control translation or by binding to splicing factors to modulate splicing, and at the post-translational level. For this last function, it is reported that *HOTAIR* could serve as a ubiquitination protein and subsequent degradation platform [56].

Most lncRNAs possess miRNA recognition elements (MREs), suggesting that the transcription of some miRNAs is regulated by lncRNAs and some lncRNAs are involved in synthesis, maturation, and degradation of miRNAs [57]. Many studies reported the interaction between *HOTAIR* and microRNAs highlighting that these interactions are able to modulate different cellular processes [58,59].

During embryogenesis, *HOTAIR* is involved in the development of the lumbosacral region, and its activity is closely linked to the recruitment of PRC2 to its targeted HOX D genes for their repression [52].

Several studies have pointed out the role of *HOTAIR* as a cell cycle-associated gene. HOX transcript antisense RNA (*HOTAIR*) promotes the cell cycle passing through the restriction point during the G1 phase by regulating CDK4/6-cyclin D and the Rb-E2F pathway [60].

In the last ten years, the aberrant *HOTAIR* expression in the majority of solid cancers has been reported, underlining its main role in modulating tumor initiation, growth, angiogenesis, progression, recurrence, drug resistance, and poor prognosis [53,61,62]. In urological cancers, *HOTAIR* overexpression is able to increase prostate cancer cells growth and invasion by binding androgen receptor (*AR*) protein and blocking its degradation [63]. In bladder cancer patients, *HOTAIR* is an independent prognostic factor of tumor recurrence [64]. It is also involved in chemo sensitivity to doxorubicin [65] and can be detected in the urine of bladder cancer patients [66]. In gynecological tumors, *HOTAIR* is overexpressed in epithelial ovarian cancer tissues and correlates with International Federation of Gynecology and Obstetrics (FIGO) stage, histological grade of the tumor, lymph node metastases, and poor survival [67]. In cervical cancer tissues, *HOTAIR* is associated with clinical-pathological features, lymph node metastases, and prognosis [68]. HOX transcript antisense RNA (*HOTAIR*) is also able to interact with different mRNAs in cervical cancer cells modulating cell growth and proliferation [69]. Moreover, the detection of circulating levels of *HOTAIR* is strongly associated with advanced tumor disease, lymph nodes metastases, and poor survival in cervical cancer patients [70]. Aberrant *HOTAIR* expression in endometrial carcinoma correlates with grade, lymph nodes metastases, and poor prognosis [71], and it is associated with cisplatin resistance acquisition [72]. In gastrointestinal tract tumors, *HOTAIR* upregulation appears as an important marker in colorectal cancer [73] and gastric cancer [74], showing a strong relation with stage, lymph nodes, distant metastases, and worse survival. In gastric cancer, *HOTAIR* has been detected in patients’ plasma, and its circulating level is able to predict which patient can benefit from fluorouracil and platinum combination therapy [74]. In liver cancer, *HOTAIR* is overexpressed and strongly correlates with clinical-pathological features, and tumor progression [75]. In addition, *HOTAIR* silencing increases chemotherapy sensitivity to cisplatin and doxorubicin hepatocellular carcinoma patients [76]. In oral cancers, *HOTAIR* overexpression has been described in laryngeal squamous cell carcinoma (LSCC) and is associated with histopathological grade and stage [77]. Also, in LSCC cells, *HOTAIR* is involved in the modulation of sensitivity to cisplatin [78]. In lung cancer, aberrant expression of *HOTAIR* correlates with advanced stage, lymph nodes metastases, and poor prognosis [79]. A higher *HOTAIR* expression is also strongly associated with cisplatin resistance in non-small cell lung cancer (NSCLC) patients [80]. Circulating *HOTAIR* has been detected in lung cancer plasma, and it appears to be associated with clinical-pathological features of the patients [81].

Many studies have highlighted the role of *HOTAIR* also in tumor microenvironment (TME) intracellular signaling. In TME, *HOTAIR* is able to modulate different molecular pathways involved in tumor phenotype modifications during metastatic progression [82].

## 4. HOTAIR’s Role in Breast Cancer

HOX transcript antisense RNA (HOTAIR) belongs to the first lncRNAs which have aberrant expressions that have been identified to associate with BC progression [61]. It is able to interact with the main molecular pathways involved in BC carcinogenesis. Estradiol can regulate *HOTAIR* expression in ER+ BC cells for the presence of several EREs elements in its promoter [83]. Estradiol agonists, bisphenol-A and diethylstilbestrol are able to stimulate *HOTAIR* expression in in vitro and in vivo BC models [84]. Moreover, both *HOTAIR* and breast cancer gene 1 (*BRCA1*) are able to bind the subunit of *EZH2* (Enhancer of Zeste 2 Polycomb Repressive Complex 2 Subunit) coordinating the PRC-dependent epigenetic regulation of the chromosome [85]. Breast cancer gene 1 (*BRCA1*) is able to inhibit the binding of *EZH2* to *HOTAIR* and its transfer on the promoter of PRC2 target gene *HOXA9* in human BC cells and fibroblasts [85]. The promoter of the *HOTAIR* gene can also be bound by *IRF1* (interferon regulatory factor-1) able to induce its inhibition in BC cells [86]. It is known that *HOTAIR* is also associated with an aberrant DNA methylation profile in cancer [87]. In BC, the combination of *HOTAIR* overexpression and methylation status represents an important predictor of poor prognosis [88,89].

### 4.1. HOTAIR’s Role in BC Metastatic Progression

Early studies highlighted the aberrant expression of *HOTAIR* in primary BC tumors with high metastatic potential and poor survival, suggesting *HOTAIR* as a powerful predictor of BC tumor progression [90]. Further studies have then proved contrasting results about the role of *HOTAIR* in prediction of metastatic risk in the different molecular subtypes of BC [91]. Some authors suggested that *HOTAIR* is an independent predictor of metastasis in ER+ patients but not in ER− BC patients [92]. On the contrary, other studies showed that the upregulation of *HOTAIR* can be considered a marker of metastatic progression only in ER− BC patients [91]. These latest data have also been confirmed by in vitro investigations pointing out aberrant expression of *HOTAIR,* in particular in basal-like BCs [93]. A recent study analyzed the in situ expression of *HOTAIR* in a large case series of TNBC patients and showed that high *HOTAIR* expression in tumor tissues is strongly correlated with lymph node metastasis, and it is directly associated with androgen receptor (AR) expression therefore potentially involved in the regulation of the AR pathway [94].

### 4.2. HOTAIR’s Role in Epithelial-Mesenchymal Transition

Many studies have demonstrated that *HOTAIR* is also a critical modulator of EMT in BC. The treatment of HCC1954 BC cells with TGF-B1 leads *HOTAIR* upregulation and modulates the EMT process. This condition is reversed by induced downregulation of *HOTAIR* with a consequent reduction in the ability to form colonies [95]. Recently, it was shown that Cancer-associated fibroblasts (CAFs) are able to promote BC metastasis via paracrine *TGF-B1*. The CAF-conditioned media of MCF7 and MDA-MB-231 BC cells strongly increases *HOTAIR* expression promoting EMT [96]. Autophagy is also strongly involved in the modulation of EMT. HOX transcript antisense RNA (*HOTAIR*)-mediated autophagy could be a critical step in BC progression thanks to its ability to induce upregulation of metalloproteinases (MMPs) and B-catenin [83]. The modulation of the EMT process as well as the consequent induction of metastatic processes is also strongly influenced by the activity of a series of microRNAs, especially in BC. The downregulation of miR-7 in BC patients is strongly associated with BC cancer stem cells and correlates with *HOTAIR* expression. The knockdown of *HOTAIR* leads to miR7 upregulation and reverts EMT and BC cancer stem cells proliferation [97]. A recent study has highlighted that *HOTAIR* is able to induce BC evolution by increasing the *Bclw* gene, belonging to the B-cell lymphoma 2 (bcl-2) family, via sequestering miR-206 at the post-transcriptional level [58]. Moreover, *HOTAIR* is able to physically interact with the miR34 promoter to silence miR34a in cancer stem cells (CSCs) from BC cells [98]. Recently, Han et al. [59] showed that delphinidin, an anthocyanidin, is able to suppress BC progression by upregulating the miR34a inhibition of *HOTAIR* and suppressing EMT through the downregulation of MMPs and the beta-catenin signaling pathway.

### 4.3. HOTAIR’s Role as a Circulating Marker

The great diagnostic and prognostic potential of *HOTAIR* has also been supported by its detection in the blood of BC patients [99]. The circulating DNA level of *HOTAIR* from BC patients strongly correlates with the clinical stage, regardless of the molecular subtype [100]. Zhang et al. [101] showed that *HOTAIR* expression, analyzed in 148 plasma samples from BC patients, significantly correlates with *ER* and *HER2* expression and with lymph node metastasis. In post-operative BC patients, a substantial reduction of its circulating level has been described [101]. More recently, Tang et al. [102] showed that serum exosomal *HOTAIR* is a potent predictor of poor survival and drug response in BC patients, regardless of the molecular subtype [102].

## 5. *HOTAIR* in Breast Cancer Therapeutic Resistance

One of the main problems in breast cancer therapy is the establishment of an intrinsic or acquired resistance to treatment. Resistance to anti-tumor therapies can be linked to a variety of different factors such as genetic mutations, increased drug efflux, tumor heterogeneity, altered crosstalk between tumor cells and environmental factors or epigenetic changes related to the aberrant activity of many ncRNAs [103,104,105,106,107,108,109]. However, the knowledge of the mechanisms of resistance to the routine therapeutic agents in BC remains widely unknown. Long non coding RNA (LncRNAs) seem to be largely involved in drug responses for their ability to modulate the expression pattern of many oncogenes and oncosuppressor genes [110,111,112,113,114]. HOX transcript antisense RNA (*HOTAIR*) aberrant expression has been widely described as a marker of drug resistance in different solid tumors [61,115]. It can be involved in different resistance mechanisms related to the main routine treatments including radiotherapy, chemotherapy, and target therapies.

### 5.1. Radiotherapy Resistance

In cervical cancers, *HOTAIR* overexpression is able to induce radio-resistance via inhibiting p21, and its knockdown, by upregulating p21, increases the radio-sensitivity of cervical cancer cells [116]. Moreover, *HOTAIR* silencing is able to increase radio sensitivity and influence autophagy in prostate cancer cells [117]. Radiotherapy is the leading therapeutic strategy for inoperable and locally advanced breast cancers. Zhou et al. [118] investigated *HOTAIR* gene expression in five breast cancer tumor cell lines showing that the upregulation of *HOTAIR* in MDA-MB231 cells accelerates cell proliferation and enhances the resistance to radiotherapy.

To investigate the mechanism controlling *HOTAIR* induced radio-resistance, the expression of *HOXD10*, the translation of which is repressed by *HOTAIR* [52] contributing to the acquisition of metastatic phenotypes, was analyzed. For the same purpose, the expressions of p*BAD* (Bcl2-associated agonist of cell death) involved in apoptotic pathway, and pAKT, involved in the cell proliferation pathway, were evaluated. The results showed that *HOTAIR* promotes the proliferation of BC cells during radiation therapy by targeting *HOXD10* and the PI3K/AKT-BAD pathway [118].

**Figure 1:**
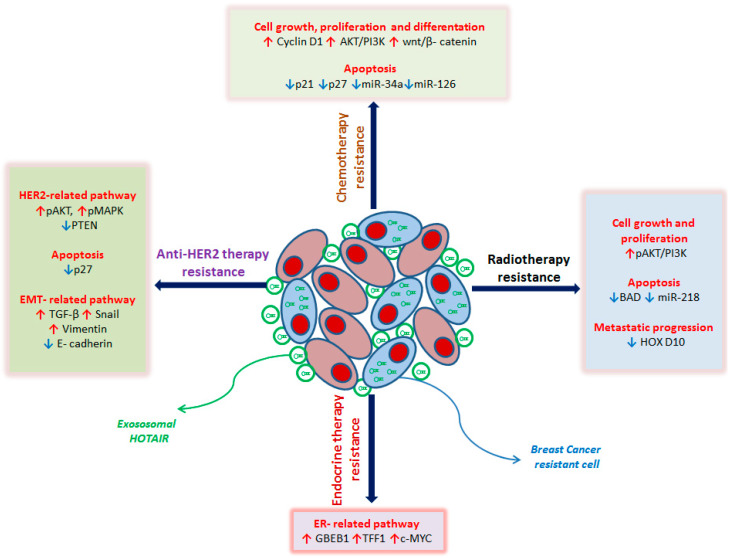
Schematic representation of *HOTAIR* role in breast cancer (BC) drug resistance mechanisms with details of the main molecular pathways involved. In anti-HER2 treatment-resistant BC cells, the overexpression of *HOTAIR* leads to: (i) deregulation of HER2-related genes by upregulating the signal transduction pathway PI3K-Akt and downregulating the tumor suppressor gene *PTEN* with the consequent increase in proliferation, cell growth, and survival; (ii) inhibition of apoptosis by the downregulation of cyclin-dependent kinase inhibitor p27; (iii) induction of EMT by *TGF-β, Snail* and *Vimentin* upregulation, and decrease in *E-cadherin* expression. In endocrine therapy resistant BC cells, the overexpression of *HOTAIR* leads to the repression of ER and the activation of ER-responsive genes, such as *GREB1, TFF1*, and *c-MYC*, promoting cell proliferation. In BC radio-resistant cells, the overexpression of *HOTAIR* leads to: (i) promotion of cell growth and proliferation by upregulation of the PI3K-Akt pathway; (ii) blockage of apoptosis by downregulating the pro-apoptosis gene *BAD* and miR-218, normally involved in the repair of radiation-induced DNA damage; (iii) induction of metastatic spread by silencing of *HOXD10*, a metastasis suppressor gene. In chemo-resistant BC cells, the overexpression of *HOTAIR* leads to: (i) promotion of cell growth, differentiation, and proliferation by upregulating Cyclin D1, the PI3K-Akt pathway, and the wnt/β-catenin pathway; (ii) inhibition of apoptosis by downregulating cyclin-dependent kinase inhibitors *p21* and *p27*, miR-34a and miR-216, both involved in promoting programmed cell death. The red arrows indicate the upregulated genes, the blue arrows the downregulated genes. HER2: human epidermal growth factor receptor 2, PI3K: Phosphoinositide 3-kinases, Akt: protein kinase B, PTEN: Phosphatase and tensin homolog, TGF-beta: Transforming growth factor beta 1, EMT: epithelial–mesenchymal transition, ER: Estrogen Receptor, GREB1: Growth Regulating Estrogen Receptor Binding 1, TFF1: Transcription Termination Factor 1, c-MYC: myelocytomatosis viral oncogene homolog, BAD: BCL2 antagonist of cell death, HOXD10: Homeobox D10.

Lately, it has been described that the expression of *HOTAIR* increases following ionizing radiation treatment. HOX transcript antisense RNA (*HOTAIR*) knockdown results in slower proliferation of BC cells, DNA damage accumulation, cell cycle arrest in the G2/M phase, and an increase in radiation-induced cell apoptosis. The radiosensitizing effects of *HOTAIR* silencing are related to the recruitment of miR-218, a ceRNA of *HOTAIR*, involved in repairing radiation-induced DNA damage and in apoptosis [119] (Figure 1).

### 5.2. Endocrine Therapy Resistance

The antagonist of the estrogen receptor Tamoxifen is the most commonly used drug for ER+ BC patients, but the acquired resistance to the treatment represents the most important limitation for its use [120]. Xue et al. showed that 37 lncRNA genes are repressed by estrogen and up regulated in tamoxifen-resistant MCF7 cells. *HOTAIR* is the main upregulated lncRNA in tamoxifen-resistant breast cancer and it is able to interact with ER, repressing it, but enhancing its transcriptional activity also in the absence of ligand. *HOTAIR* overexpression is able to induce ER-target gene expression such as GREB1, TFF1 and c-MYC in the absence of estrogen.

Knockdown of *HOTAIR* strongly decreases tamoxifen-resistant MCF7 cell growth and inhibits the colony-formation abilities. These data suggest that *HOTAIR* is involved in tamoxifen-resistant cell growth and that this drug resistance may be reverted by targeting *HOTAIR* [121] (Figure 1).

Aromatase Inhibitors (AI) act blocking the enzyme aromatase, involved in the biosynthesis of estrogen reducing the growth of hormone-receptor-positive BC cells. AI are mainly used in postmenopausal women in whom it has better therapeutic effects than tamoxifen [122].

Preliminary data performed on a large series of hormone receptor-positive early BC patients treated with AI, directly or after tamoxifen switch, showed that *HOTAIR* overexpression strongly correlates with clinic-pathological parameters, survival and AI resistance [123].

### 5.3. Anti-HER2 Therapy Resistance

Trastuzumab is a humanized monoclonal antibody that binding HER2 receptor suppresses the formation of HER2 dimer interfering with downstream signaling pathways and promotes the inhibition of cell proliferation and apoptosis [122]. Resistance to trastuzumab is one of most clinic issues for HER2+ BC patients [124]. A more recent study has showed that, in trastuzumab-resistant BC cell line, *HOTAIR* is overexpressed. In these cells, *HOTAIR* promotes the transition of tumor cells from G1 phase to S phase and inhibits the apoptosis.

To define the molecular mechanisms underlying the trastuzumab resistance mediated by *HOTAIR*, HER2 receptor signaling pathway related, PI3K/AKT/mTOR and MEK/MAPK, have been analyzed in the sensitive and resistant BC cells. In resistant cells, *HOTAIR* overexpression is associated with the upregulation of p-AKT, p-MAPK and CyclinD1 and with the downregulation of tumor suppressor gene *PTEN* and cyclin-dependent kinase inhibitor *P27*, involved in the block of G_1_/S-phase transition. This leads to an increase in the cell growth, proliferation, survival and apoptosis.

The knockdown of *HOTAIR* leads to the downregulation of p-AKT, p-MAPK and CyclinD1, and upregulation of *PTEN* and *P27*. This silencing is able to sensitize BC cells to trastuzumab blocking cell division at G0/G1 phases and promoting apoptosis.

Moreover, in the resistant cells, the transcription and translation of *TGF-β*, *Snail* and *Vimentin* are up regulated while *E-cadherin* is downregulated, promoting EMT. *HOTAIR* silencing reverts these results [125] (Figure 1).

### 5.4. Chemotherapy Resistance

Cytotoxic chemotherapy is largely used in routine therapeutic schemes both in advanced and early BC stages [122]. In particular, anthracyclines (mainly epirubicin and doxorubicin) are considered standard adjuvant therapy for patients with high-risk early BC [126]. Along with anthracyclines, which are extremely cardiotoxic, taxanes (mainly docetaxel and paclitaxel) are the most active cytotoxic drugs in BC. [127]. Furthermore, fluorine derivatives (5-fluorouracil and capecitabine), methotrexate, vinorelbine, gemcitabine, and platinum derivatives (mainly cisplatin and carboplatin) represent the therapeutic alternatives for BC patients, alone or in combination with other drugs [128].

HOX transcript antisense RNA (*HOTAIR*) has been described as being involved in doxorubicin resistance in gastric and bladder cancer [65,129], in taxanes resistance in gastric cancer (GC) and laryngeal squamous cell carcinoma [129,130], and in 5-fluoruracil resistance in GC and colorectal cancer [74,131]. Regarding platinum derivatives, *HOTAIR* is involved in modulating resistance to carboplatin in ovarian cancer [132]. In addition, many studies have shown that *HOTAIR* plays a key role in cisplatin resistance. HOX transcript antisense RNA (*HOTAIR*) upregulation can promote the resistance of lung cancer cells to cisplatin by downregulating p21, involved in the block of G_1_ and G_2_ cell cycle phases [80,133]. In ovarian cancer cells, *HOTAIR* induces cisplatin resistance by activating the wnt/β-catenin pathway, involved in key cellular functions including proliferation, differentiation, migration, genetic stability, apoptosis, and stem cell renewal. Pre-treatment with the wnt/β-catenin inhibitor, XAV939, and *HOTAIR* knockdown increases the sensitivity of cisplatin by inhibiting cisplatin-induced autophagy [134]. HOX transcript antisense RNA (*HOTAIR*) overexpression is related with cisplatin resistance in gastric cancer [135,136] and in oral cancers [137]. In gastric cancers, *HOTAIR*-induced cisplatin resistance is mainly related with the activation of the PI3K/Akt signaling pathways via miR-34a and miR-126, both involved in the modulating the apoptotic process [135,136].

Despite the numerous contributions for other solid tumors, in BC, very few studies have been performed to verify the role of *HOTAIR* in cytotoxic chemotherapy resistance and are mainly aimed at defining the predictive value of its circulating levels. Tang et al. [102] analyzed circulating *HOTAIR* levels in the serum of 112 breast cancer patients before neoadjuvant chemotherapy (NAC) treated with different cytotoxic drugs to evaluate its predictive value. The study showed that high circulating *HOTAIR* levels strongly correlate with poor response to NAC [138]. Furthermore, a recent study showed that serum exosomal *HOTAIR* levels in BC patients 3 months after surgery are significantly reduced compared to levels before surgery, and a high level correlated with poor neoadjuvant chemotherapy [102].

Many natural products derived from dietary sources can be used in BC treatment [139]. Delphinidin is one of the main anthocyanidins and has strong anti-cancer properties, and it is able to suppress tumor transformation in breast cancer cells [140]. Delphinidin is able to downregulate *HOTAIR* and simultaneously upregulate miR-34a, inducing apoptosis, in BC cells. Moreover, delphinidin treatment significantly decreases β-catenin, glycogen synthase kinase-3β (*Gsk3β*), c-*Myc*, *cyclin-D1,* and matrix metalloproteinase-7 (*MMP-7*) expression. HOX transcript antisense RNA (*HOTAIR*) overexpression, in turn, can block the effect of delphinidin on the miR-34a and Wnt/β-catenin signaling pathway in MDA-MB-231 cells, suggesting that delphinidin may potentially suppress breast carcinogenesis through the HOTAIR/miR-34a axis [59] (Figure 1).

## 6. *HOTAIR* as Therapeutic Targets in Breast Cancer

In recent years, having been strongly validated the clinic-diagnostic capabilities of lncRNAs, many therapeutic strategies have been suggested for targeting lncRNAs [141]. Some lncRNAs have already been validated as potential therapeutic targets, with very encouraging results obtained on cell and animal models. The interference systems used can be of different types even if lncRNAs silencing strategies must necessarily take into account their cytoplasmic or nuclear sub-location [142,143,144]. For nuclear lncRNAs, the best approaches are represented by anti-sense-oligos (ASOs) targeting, while for knockout of cytoplasmic lncRNAs, the best results have been obtained with small interfering RNAs (siRNAs). [145]. The ASOs are short (8–50 nt) single-stranded DNAs or RNAs designed considering target RNAs specific sequences. The ASOs are characterized by high stability and sensitivity and act by binding and degrading target RNAs. Ribonuclease (RNAse) H1 recognizes the DNA:RNA heteroduplex and cuts RNA molecules [146,147]. Several studies reported the use of the ASO approach in the silencing of lncRNAs in different tumors [148]. Small interfering RNAs (siRNAs) are short (19–30 nt) double-stranded RNAs able to target RNA molecules via complementary to sequence. Small interfering RNAs (siRNAs) act through the association with RNA-induced silencing complex (RISC) complex leading to argonaute mediated degradation as a result of perfect sequence similarity [149]. Small interfering RNAs (siRNAs) can represent an easy method for both the design and synthesis and have high silencing efficiency against numerous lncRNAs. For this reason, it represents, together with ASOs, the most used method in functional studies to validate lncRNAs’ role in cancer [150]. However, other strategies could be developed to block lncRNA activity: (i) Aptamers are short DNA or RNA oligonucleotides or peptides with a stable three-dimensional structures. Integration of aptamers into cancer cell genomes could produce functional aptamers able to target both nuclear and cytoplasmic lncRNAs [151]. (ii) Ribozymes are RNA molecules involved in intracellular catalytic functions, able to degrade different RNA molecules. They could be artificially synthesized to target lncRNAs [152]. (iii) Small molecules, such as tetracyclines or aminoglycosides that are able to degrade bacterial ribosomes, could be synthetically developed for lncRNAs targeting [153]. (iv) microRNAs’ induction to target lncRNAs could also be a valid strategy, having been largely demonstrated that microRNA-lncRNA interaction could inhibit lncRNAs function. The targeting strategy could be developed by analyzing the putative regulating microRNAs of the lncRNA to be silenced [154].

**Figure 2:**
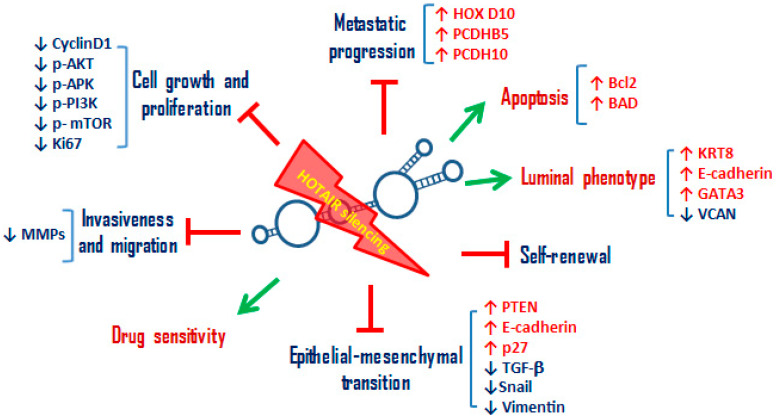
Schematic representation of the main cellular processes inhibited or activated by the silencing of *HOTAIR* in BC cells. *HOTAIR* knockdown in BC cells leads to the promotion of: (i) sensitivity to radiotherapy, chemotherapy, hormonal therapies and anti-HER2 therapies; (ii) luminal phenotype acquisition with the upregulation of luminal cytokeratins (KRT8), of cell adhesion molecule E-cadherin, and of transcription factor GATA3, responsible of luminal epithelial differentiation in the adult mammary gland. Furthermore, *HOTAIR* silencing in BC cells leads to the inhibition of: (i) cell growth and proliferation by downregulating the main signaling pathways involved in these cellular processes, such as PI3K/AKT/mTOR and MAPK/ERK pathway, cyclin D1 and proliferation index Ki67; (ii) invasion and migration by downregulation of metalloproteinases; (iii) EMT by upregulating epithelial markers, such as *E-cadherin*, downregulating mesenchymal markers, such as *Vimentin*, and TGF-beta signaling; (iv) self-renewal, reducing colonosphere and mammosphere formation; (v) metastatic progression by upregulating metastasis suppressor genes, such as *HOXD10, PCDHB5*, and *PCDH10*. The green arrow indicates the activated processes, the symbol † the inhibited ones. KRT8: Keratin 8, GATA3: GATA Binding Protein 3, mTOR: mammalian target of rapamycin, MAPK: mitogen-activated protein kinase, ERK: Extracellular regulated kinases, PCDHB5: Protocadherin Beta 5, PCDH10: Protocadherin 10.

Regarding *HOTAIR* silencing, the most used experimental approach is siRNA, able to deplete *HOTAIR* molecules both at the cytoplasmic and nuclear level [155]. The first knockout studies of *HOTAIR* have been performed on BC cells [90]. Gupta et al. [90] examined the effects of manipulating *HOTAIR* levels in several breast cancer cell lines. In particular, its silencing by siRNAs in MCF7, a cell line that expresses endogenous *HOTAIR*, decreases its capacity to invade Matrigel, a basement-membrane-like extracellular matrix [90]. Knockout *HOTAIR* studies made it possible to validate the main role of *HOTAIR* in the modulation of cell proliferation, invasion, migration as well as in the apoptotic processes in BC models [58,83,156] (Figure 2). Bhan et al. [157] used a synthetic small interfering sense (siSENSE) oligonucleotide DNA complementary to HOTAIR transcript. The normal phosphodiester bonds of the HOTAIR-siSENSE DNA molecule were replaced with phosphorothioate linkage to minimize the nuclease digestion and enhance its in vivo stability. The HOTAIR siSENSE is able to silence specifically and effectively *HOTAIR* transcript levels in a dose-dependent manner. HOX transcript antisense RNA (*HOTAIR*) silencing leads to apoptosis in MCF7 BC cells though upregulation of *Bcl2* and *BAD* expression. Moreover, *HOTAIR* knockdown induces upregulation of its target genes *HOXD10* and *PCDHB5* [157] (Figure 2). These data on MCF7 BC cells have been confirmed by the siRNA downregulation of *HOTAIR* or *EZH*2, a member of PRC2. This silencing is able to repress BC cells’ proliferation, invasion, and migration and, at the same time, to promote apoptosis [158]. HOX transcript antisense RNA (*HOTAIR*) siRNA in MCF7 BC cells is also able to increase mRNA levels of the luminal markers such as *GATA3, KRT8*, and *E-cadherin* and to reduce the basal marker as *VCAN* (Figure 2). HOX transcript antisense RNA (*HOTAIR*) expression in BC cells can be enhanced through prolonged and progressive exposure to TNF-α, a cytokine produced by the tumor microenvironment. The inhibition of p38 and SRC kinases, two mediators of the cell responses to TNF-α, can decrease *HOTAIR* expression and restore the expression of *E-cadherin* and *KRT8* in MCF-7 cells [93]. HOX transcript antisense RNA (*HOTAIR*) silencing in BC cells also shows a great impact on the modulation of EMT processes and in the self-renewal capacity of BC CSCs, being the majority of EMT/stemness genes regulated by *HOTAIR* (Figure 2). HOX transcript antisense RNA (*HOTAIR*) silencing leads to a downregulation of *TGF-β*, *Snail, Vimentin, p-AKT, p-APK*, and *CyclinD1* and an upregulation of *E-cadherin, PTEN*, and *P2*7, causing the inhibition of EMT in BC cells [159]. HOX transcript antisense RNA (*HOTAIR*) knockdown also leads to a strong reduction in colonosphere and mammosphere formation, suggesting its main role in the maintenance of the CSC phenotype in BC cell lines [95] (Figure 2). Deng et al. [98] further confirmed these observations though HOTAIR silencing with lentivirus LV-HOTAIRKD, highlighting a strong inhibition of proliferation, colony formation, migration, and self-renewal capacity of an enriched of CSCs MCF7 cell lines [98].

Due to the fact of its important role in therapeutic resistance mechanisms, several studies have reported that *HOTAIR* downregulation is able to make BC cells sensitive to different therapeutic treatments (Figure 2). Hu et al. [119] showed that silencing of *HOTAIR* in MCF7 BC cells is able to reduce cell survival inducing apoptosis in response to ionizing radiation. Moreover, in *HOTAIR* knockdown cells ionizing radiation induces more DNA damage and cell cycle arrest than in control cells [119]. In a doxorubicin-resistant BC cell line (DOXR-MCF-7), *HOTAIR* silencing decreases cell proliferation and induces apoptosis in BC cells reducing doxorubicin resistance and simultaneously determines a reduction of *PI3K, AKT*, and *mTOR* phosphorylation inhibiting the molecular pathway [160]. Similarly, in a trastuzumab-resistant breast cancer cell line SK-BR-3-TR, knockdown of *HOTAIR* sensitizes BC cells to trastuzumab [125].

Although the majority of the functional studies on *HOTAIR* performed its direct inhibition by siRNA methods, the ability to translate these methods in clinical practice is complicated. In fact, most lncRNAs are preferentially expressed in the nucleus and can be integrated into more complex structures that are not easily accessible. Therefore, small molecules designed to specifically interfere with conserved RNA structures and to block RNA protein complexes may be useful. Two very recent studies showed how *HOTAIR* activity can be efficiently blocked by molecular interference in HOTAIR/EZH2 scaffold interaction [161,162]. HOX transcript antisense RNA (*HOTAIR*) promotes BC progression in a PRC2-dependent manner [90,163] by recruiting and binding *EZH2*, the catalytic subunit of PRC2, to silence target genes [164]. Ren et al. [161], analyzing the structure and function of *HOTAIR*, identified a small molecule, named AC1NOD4Q, able to inhibit HOTAIR/PRC2 complex interaction [161]. The minimal 5′ domain of *HOTAIR* required for PRC2 binding is 212–300 nt. Using a 3D modeling prediction, it turned out that this domain contains several hairpin loop structures and serves as a target for small molecule intervention. By performing in silico high-throughput screening, Ren et al. [161] highlighted that AC1NOD4Q (ADQ) binds to a specific *HOTAIR* micro-domain (36G46A) and induces strong molecular inference in the scaffold interaction between PRC2 complex and *HOTAIR*. Its validation on cell and animal models has provided encouraging results. In ADQ-treated MDA-MB-231 cells, the migration and invasion proprieties of BC cells are strongly reduced by cell adhesion molecules and EMT biomarkers downregulation. An MDA-MB-231 orthotopic tumor transplantation model in nude mice as performed to test ADQ treatment. A significant reduction of tumor growth and lung metastatic nodules was detected in ADQ-treated mice. Moreover, the analysis by situ hybridization and immunofluorescence of the HOTAIR/PRC2 interaction in ADQ-treated mice tissues clearly highlights that ADQ treatment reduces and relocates *EZH2* signals from nucleus to cytoplasm with *HOTAIR* disassociation. This is the first study demonstrating that ADQ is able to efficiently block HOTAIR/EZH2 interaction and recruitment of *EZH2* to target genes [161].

Similarly, another molecule capable of selectively inhibiting HOTAIR–EZH2 interaction, named AC1Q3QWB, has recently been validated in BC cells and animal models [162]. AC1Q3QWB treatment inhibits tumor cell growth and metastasis in vivo and at the same time leads the upregulation of APC2, HOXD10, PCDH10, HOTAIR–PRC2 target genes, downregulation of β-catenin, tumor cell proliferation antigen (*Ki-67*), and EMT marker such as *Vimentin*. Moreover, Li et al. [162] have also shown that the combination of a low dose of AC1Q3QWB with 3-deazaneoplanocin A (DZNep), an *EZH2* inhibitor that induces the degradation of PRC2 by impairment of SAH (S-adenosyl-l-homocysteine), already largely used in different tumors, exhibits a marked anti-tumor activity, in orthotopic BC models, superior to the agents used alone [162].

## 7. Conclusions and Perspectives

The use of different silencing systems for *HOTAIR* has allowed the obtention of encouraging results. Most of the studies on cellular and animal models have been carried out using siRNA methods. Despite their great usefulness and the easy use to understand functional mechanisms which underlie the aberrant activity of *HOTAIR* in BC cells, these silencing systems have not yet been contemplated for clinical studies. In fact, the transition from preclinical to clinical studies is quite difficult because, while in vitro models are easy to apply but have limited clinical relevance, in vivo models have higher clinical relevance, but they are expensive and challenging to conduct. To date, only few therapeutic miRNAs have successfully moved into clinical trials due to the enormous difficulty in assessing toxic and off-targets effects and in generating a high stability and successful delivery system [165].

More recently, a detailed analysis of the structure and functional mechanisms of ncRNAs (interaction with the target genes) has allowed the development of a computer-aided structure-based drug design method. This approach could efficiently screen the potential compounds in the shortest possible time, allowing optimal selection of new target compounds [161,162]. A series of compounds specifically capable of blocking the activity of some ncRNAs are already used in clinical practice. For example, some small molecules such as kanamycin, an antimycobacterial drug mainly used to treat tuberculosis, is capable of binding pre-miRNAs inhibiting DICER-mediated miRNA processing [166]. The recent identification of small molecules able to block *HOTAIR* activity by interfering with HOTAIR/EZH2 scaffold interaction are offering new strategies for its inhibition that may be easier to use in human. Enhancer of zeste 2 polycomb repressive complex 2 subunit (EZH2) inhibitor compounds, such as DZNep, have been previously suggested as potential drugs for clinical usage in solid tumors [167]. In BC preclinical models, the combination of DZNep with a small-molecule involved in the block of HOTAIR–EZH2 interaction, greatly enhances the antitumor activity [162].

Although there are still no clinical studies that can prove their usefulness in BC patients, these small molecules, being also easily synthesizable for large-scale manufacturing, are proving to be powerful anticancer agents and could represent the optimal system to block *HOTAIR* activity.

In conclusion, *HOTAIR* plays a key role in BC tumor progression, being able to modulate, directly or indirectly, crucial cell processes such as growth, proliferation, invasiveness, EMT, self-renewal, metastatic spread, and drug resistance. For this reason, although no relevant studies on drugging lncRNAs have been reported, the possibility of interfering with *HOTAIR* activity could represent an important tool for defining innovative therapeutic strategies in BC, mainly oriented for the reversal of drug resistance.

## Figures and Tables

**Table 1:** Main lncRNAs (long non-coding RNAs) involved in BC (breast cancer) progression.

LncRNA	Expression	Activity	Drug Resistance	References
*H19*	Upregulated	Promoting tumor growth, metastasis, poor prognosis	Endocrine therapy and chemotherapy resistance	[23,24,25,26,27]
*XIST*	Downregulated	Suppressing cell growth, migration, and invasion	Chemotherapy resistance	[28]
*BCAR4*	Upregulated	Promoting tumor growth, metastasis, poor prognosis	Endocrine therapy resistance	[30,31,32]
*CCAT2*	Upregulated	Promoting cell proliferation, migration, invasion, stem-like phenotype	Endocrine therapy resistance	[33]
*UCA1*	Upregulated	Promoting cell proliferation, migration, invasion	Endocrine therapy and trastuzumab resistance	[34,35,43]
*MALAT 1*	Upregulated	Promoting cell proliferation, migration, invasion, stem-like phenotype	-	[36,37,38]
*NEAT1*	Upregulated	Promoting tumor growth, metastasis, poor prognosis	Chemotherapy resistance	[39,40]
*LncRNA-ATB*	Upregulated	Promoting cell proliferation, migration, metastasis	Trastuzumab resistance	[41]
*GAS5*	Downregulated	Promoting apoptosis	Endocrine therapy and chemotherapy resistance	[45,52]
*AGAP2-ASI*	Upregulated	Promoting cell proliferation, migration, invasion	Trastuzumab and chemotherapy resistance	[44]
*TINCR*	Upregulated	Promoting cell proliferation, migration, invasion, suppressing apoptosis	Trastuzumab resistance	[48]
*LncRNA-ROR*	Upregulated	Promoting cell proliferation, migration, invasion	Chemotherapy resistance	[46]
*CyTOR*	Upregulated	Promoting tumor growth, metastasis, poor prognosis	Endocrine therapy resistance	[51]
*LINP1*	Upregulated	Promoting tumor growth, metastasis, poor prognosis, involved in DNA repair mechanisms	Endocrine therapy and chemotherapy resistance	[52]
